# Anion-Complementary Soft Solvation Electrolytes Stabilizing Dual Interfaces for High-Voltage Lithium Metal Batteries across Wide Temperatures

**DOI:** 10.1021/acsami.5c07417

**Published:** 2025-09-18

**Authors:** Siyu Sun, Huipeng Zeng, Baichuan Cui, Mingjia Zhi, Jing Zheng, Zhanglian Hong, Jijian Xu

**Affiliations:** † State Key Laboratory of Silicon Materials, School of Materials Science and Engineering, 12377 Zhejiang University, Hangzhou 310027, P. R. China; ‡ Department of Chemistry, 53025 City University of Hong Kong, Hong Kong 999077, P. R. China; § Department of Chemistry and Materials Science, College of Science, 74584 Nanjing Forestry University, Nanjing 210037, P. R. China; ∥ Shenzhen Research Institute, 53025 City University of Hong Kong, Shenzhen 518057, P. R. China

**Keywords:** Lithium metal batteries, Ether-based electrolyte, Electrolyte design, Solid electrolyte interphase, Wide-temperature

## Abstract

Lithium metal batteries (LMBs) face severe interfacial degradation due to uncontrolled lithium dendrite growth and electrolyte decomposition. Conventional electrolytes fail to concurrently stabilize lithium metal anodes and high-voltage cathodes owing to competing parasitic reactions. Here, we report a softly solvating electrolyte composed of 1,3-dioxane (1,3-DX) and dual salts—lithium bis­(fluorosulfonyl)­imide/lithium hexafluorophosphate (LiFSI/LiPF_6_)—that leverages anion-complementary coordination to decouple interfacial requirements. The solvent’s steric hindrance softens Li^+^–solvent interactions, enabling FSI^–^ and PF_6_^–^ to spatially separate their interfacial functions: FSI^–^ drives LiF-rich solid-electrolyte interphase (SEI) formation on Li metal, while PF_6_^–^ constructs a thin (<10 nm) cathode–electrolyte interphase (CEI) on LiNi_0.5_Co_0.2_Mn_0.3_O_2_ (NCM523) cathodes. Raman and ^17^O NMR spectra confirm suppressed solvent coordination and anion-aggregate dominance. Li||NCM523 cells utilizing this electrolyte achieve over 80% capacity retention after 100 cycles at 4.3 V, with a high average Coulombic efficiency (CE) above 99.0%. Cross-sectional scanning electron microscopy/transmission electron microscopy (SEM/TEM) reveals crack-free cathodes and robust, homogeneous SEI and CEI layers. Remarkably, the Li||NCM523 cells maintain 36.9% capacity retention of their room-temperature performance (54.7 vs 148.3 mAh g^–1^) at 0.2 C and −30 °C, highlighting the electrolyte’s low-temperature compatibility. This work establishes an anion-synergistic design strategy to reconcile bulk ion transport with dual electrodes.

## Graphical abstract

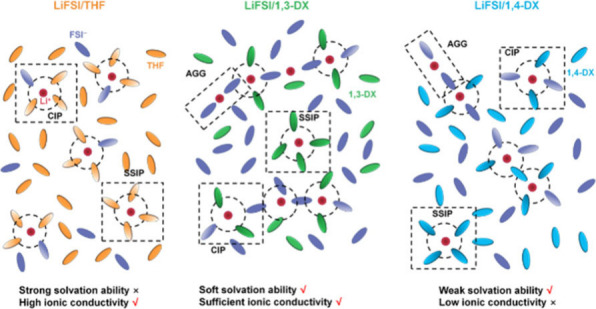

## 1 Introduction

The rapid adoption of electric vehicles (EVs), driven by global decarbonization initiatives, is constrained by their limited driving range, primarily attributed to the energy density limitations (250–300 Wh kg^–1^) of current battery technologies.^1^ This bottleneck has intensified research on alternative battery chemistries. Lithium metal batteries (LMBs) are among the most promising candidates, owing to the ultrahigh theoretical capacity (3860 mAh g^–1^) and low redox potential (−3.04 V vs SHE) of lithium metal anodes (LMAs), compared to 372 mAh g^–1^ for conventional graphite anodes.^2,3^ Consequently, LMBs are projected to achieve greater than 500 Wh kg^–1^ in energy density.^4^ However, the practical application of LMAs remains hindered by significant challenges, including ∼200% volume variation during cycling and lithium dendritic growth. These issues can induce internal short circuits and trigger thermal runaway—critical safety concerns.^5–8^

Electrolyte engineering, particularly through solvation structure modulation, has emerged as a viable approach to stabilize lithium deposition and suppress dendrite propagation. Conventional carbonate-based electrolytes typically form solid-electrolyte interphases (SEIs) dominated by organic species (e.g., ROCO_2_Li), which exhibit a low ionic conductivity (10^–8^–10^–7^ S cm^–1^) and mechanical brittleness. These properties lead to inhomogeneous lithium deposition, rapid capacity fading, and poor Coulombic efficiency (CE),^9^ while ether-based electrolytes offer improved reductive stability, enabling a high CE in Li||Cu cells.^10^ Representative solvents like 1,2-dimethoxyethane (DME) and 1,3-dioxolane (DOL) facilitate anion-derived SEIs rich in LiF, enhancing interfacial ion transport and showing promise in stabilizing LMAs.^11,12^ However, the limited oxidative stability of ethers results in severe electrolyte decomposition at high voltages, >4.3 V, particularly when paired with Ni-rich layered oxides, accelerating transition metal dissolution and structural degradation.^13^ High-concentration electrolytes (HCEs) employ elevated salt-to-solvent ratios to suppress free ether solvents and promote anion-dominated Li^+^ coordination, enabling inorganic-rich cathode–electrolyte interphase (CEI) formation.^14,15^ However, their high viscosity and poor wettability degrade interfacial kinetics.^16,17^ Localized high-concentration electrolytes (LHCEs) address viscosity through hydrofluoroether diluents but introduce environmental concerns from perfluoroalkyl substances and unresolved compatibility issues with high-voltage cathodes.^18–21^

Fluoroether-based electrolytes offer enhanced oxidative stability^22–25^ and can form uniform and stable LiF-rich SEI and CEI layers that mitigate transition metal dissolution and suppress dendrite growth.^26–32^ Despite their promise, their widespread use is hindered by toxicity concerns and complex synthesis processes. Weakly solvating electrolytes stabilize lithium deposition by forming anion-dominated solvation structures that preferentially decompose into inorganic-rich interphases, effectively suppressing dendrite growth and enabling high Coulombic efficiency.^33–36^ Additionally, the use of weak solvents can enhance low-temperature performance by reducing the desolvation energy barrier for lithium ions.^37^ However, their weak solvating power limits salt dissociation, leading to high ion-pair clustering and low ionic conductivity (<3 mS cm^–1^), which restricts high-rate capability and compatibility with high-voltage cathodes.^38,39^ Therefore, identifying an optimal solvent that addresses these multifaceted challenges is of paramount importance. An ideal electrolyte for LMBs should combine the reductive stability of ether-based solvents, enhanced oxidative stability, low viscosity, and high ionic conductivity.^40^ Inspired by our recent work, we propose the use of softly coordinating solvents to strike a balance between sufficient ionic conductivity and low desolvation energy.^41^

In this work, we report a molecular-engineered soft solvating electrolyte based on 1,3-dioxane (1,3-DX) to address key challenges in high-voltage LMBs. By systematically investigating the solvation structure and electrochemical performance, we reveal that the asymmetric oxygen geometry of 1,3-DX enables anion-dominated solvation structures, resulting in spatially decoupled interfacial reactions. The optimized electrolyte delivers exceptional cycling stability, with 80% capacity retention after 100 cycles in Li||LiNi_0.5_Co_0.2_Mn_0.3_O_2_ (NCM523, 1.63 mAh cm^–1^) cells under 4.3 V operation, coupled with suppressed cathode degradation and minimal side reactions. At −30 °C, NCM523 cathodes maintained 36.5% capacity retention (54.7 mAh g^–1^, 0.2 C) relative to room-temperature performance (148.3 mAh g^–1^, 0.2 C), demonstrating a stable operation under low-temperature conditions. This work underscores the critical role of solvent geometric asymmetry in tailoring solvation dynamics and interphase chemistry, providing a molecular engineering strategy for high-energy-density battery systems across a wide temperature range.

## 2 Result and Discussion

### 2.1 Electrolyte Design and Solvation Structures

The investigation of the electrostatic potential (ESP) distribution of these solvents—tetrahydrofuran (THF), 1,3-DX, and 1,4-dioxane (1,4-DX)—and the binding energy between lithium ions and these solvents revealed critical structure–performance relationships. As shown in Figure 1a, 1,3-DX and 1,4-DX display relatively low ESP values for the oxygen atom (indicated by red regions), suggesting weak coordination. In contrast, THF shows concentrated negative potentials at its oxygen atoms, indicating stronger coordination with Li^+^ through specific heteroatom interactions.

**Figure 1:**
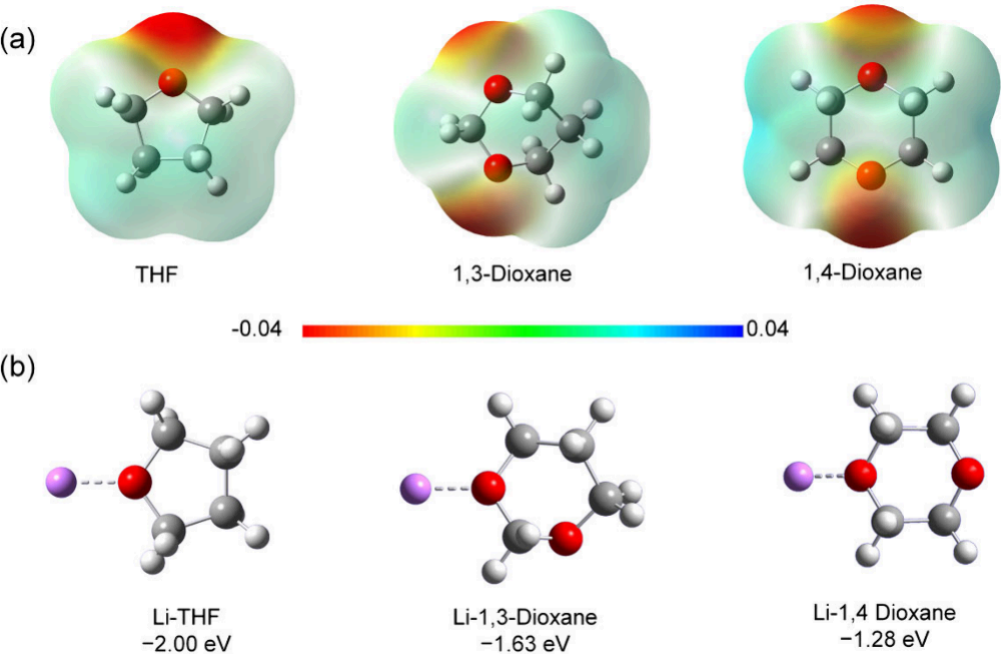
**a** Electrostatic potential mappings of THF, 1,3-DX, and 1,4-DX. The color scale indicates electrostatic potential distribution, with red and blue representing lower and higher potential, respectively. **b** Calculated binding energies of Li^+^–solvent coordination structures.

Among the tested solvents, 1,3-DX exhibits an intermediate Li^+^ binding energy (−1.63 eV), as quantified in Figure 1b, reflecting its soft coordination strength. This reduced binding energy is attributed to the low electron density of its oxygen atoms, which may alleviate local charge accumulation and facilitate easier Li^+^ desolvation. In comparison, THF shows a stronger binding energy of −2.00 eV, forming more stable solvent–Li^+^ complexes that could hinder ion desolvation during battery operation, particularly at low temperatures. While 1,4-DX’s binding energy is the lowest (−1.28 eV), this comes at the expense of ionic conductivity, as discussed in the following section.

The physicochemical properties of these cyclic ether solvents are summarized in Table S1. The observed decoupling between the dielectric constant and lithium salt solubility in 1,3-DX challenged conventional electrolyte design paradigms. While its elevated dielectric constant theoretically favors ionic dissociation, the practical LiFSI solubility is limited to 1.4 M at 25 °C. This constraint originates from molecular-level competition: the asymmetric oxygen coordination geometry created significant spatial hindrance that restricts salt accommodation, overriding the dielectric advantages. This phenomenon highlights how molecular packing efficiency, rather than bulk polarity alone, can dominate the lithium dissolution capacity in conformationally restricted solvents. Crucially, 1,3-DX demonstrates exceptional thermal stability with a melting point of −45 °C and a boiling point of 105 °C, enabling phase stability across a 150 °C span. This is a notable improvement over conventional ether solvents, which often suffer from either extreme volatility (THF) or high melting points (1,4-DX at 11.75 °C). The combination of moderate dielectric strength for sufficient ion dissociation and intrinsic molecular rigidity for limited salt dissolution positions 1,3-DX as a unique soft-solvation medium. It offers the potential to avoid concentration polarization issues while maintaining wide electrochemical windows—a critical balance rarely achieved in existing electrolyte systems.

The coordination characteristics of cyclic ether solvents were systematically investigated through Raman spectroscopy under an equivalent 1 M LiFSI concentration (Figure 2a). Comparative analysis of THF, 1,3-DX, and 1,4-DX revealed distinct Li^+^ coordination behaviors, dictated by their molecular architectures, while solvent-specific vibrations (800–1000 cm^–1^) exhibited minor shifts upon LiFSI addition, as shown in Figure S1, confirming weak solvent–Li^+^ interactions across all electrolytes: the critical differentiation emerged in the FSI^–^ coordination regime (700–760 cm^–1^). THF, a five-membered cyclic ether with a single oxygen coordination site, displayed a FSI^–^ S–N–S vibrational peak at 718.1 cm^–1^, characteristic of moderate Li^+^–anion pairing. In contrast, dioxanes exhibited blue-shifted peaks due to their oxygen spatial arrangements: 1,3-DX (730.74 cm^–1^) and 1,4-DX (731.71 cm^–1^). This 12–13 cm^–1^ upshift relative to THF originated from the cooperative electron-withdrawing effects of dual oxygen atoms in dioxanes, which strengthen FSI^–^–Li^+^ interactions through enhanced charge polarization.

**Figure 2:**
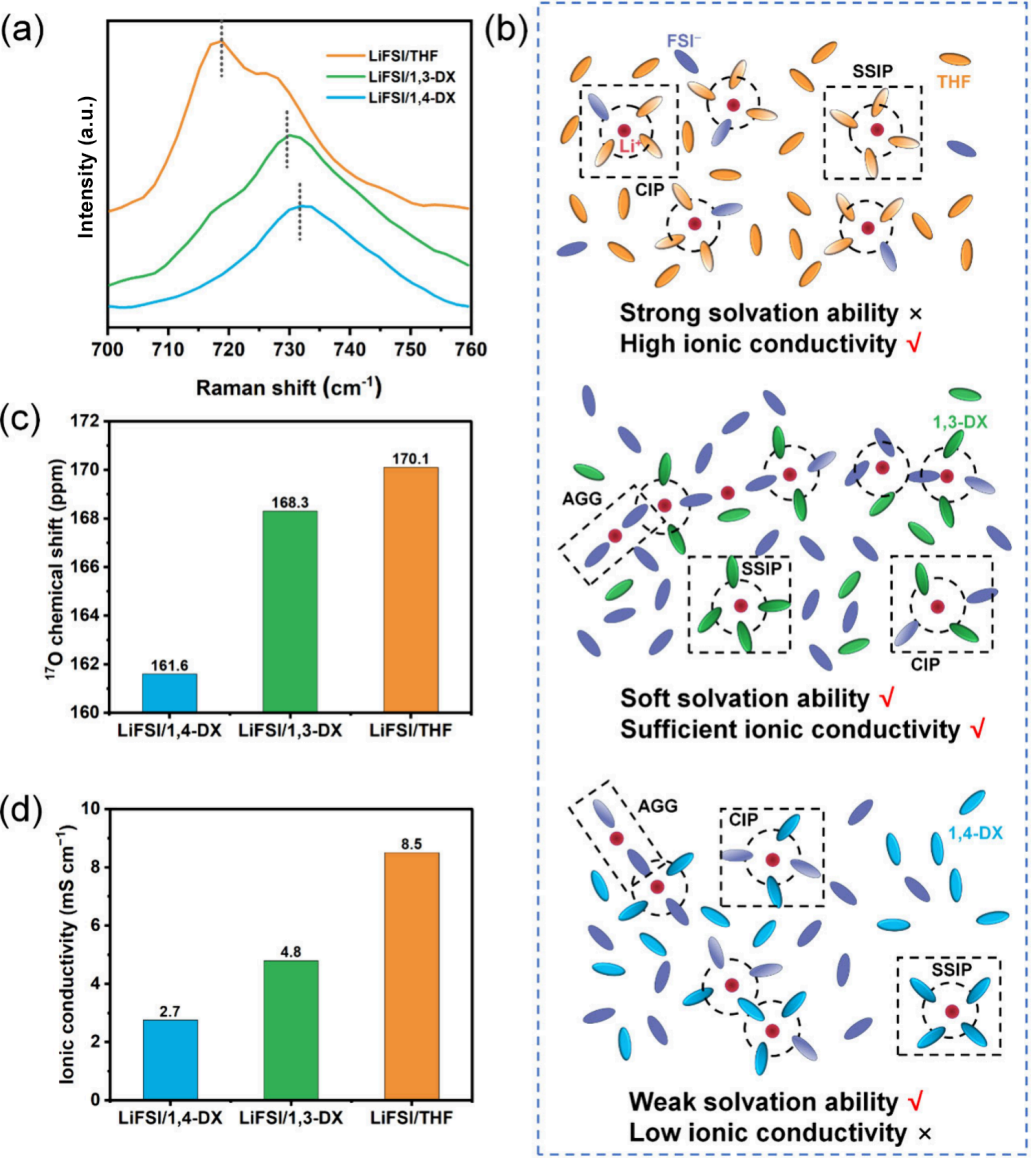
**a** Raman spectra of LiFSI-based electrolytes in THF, 1,3-DX, and 1,4-DX within the 700–760 cm^–1^ range. **b** Schematic diagram of the solvation structures in THF, 1,3-DX, and 1,4-DX electrolytes. **c** ^17^O NMR chemical shifts of FSI^–^ in THF, 1,4-DX, and 1,3-DX solvents. **d** Ionic conductivity of LiFSI-based electrolytes with THF, 1,4-DX, and 1,3-DX.

Based on the results of the Raman spectral deconvolution (Figure S2), three schematic diagrams of the solvation structures were drawn (Figure 2b). THF primarily formed solvent-separated ion pair (SSIP) and contact ion pair (CIPs) configurations with minimal aggregate (AGGs) formation, owing to its monodentate oxygen coordination. Conversely, 1,3-DX and 1,4-DX showed co-dominant CIP and AGG structures with negligible solvent-separated ion pairs.

Nuclear magnetic resonance (NMR) spectroscopy provides a direct probe of Li^+^ solvation environments by correlating the solvent electronic structure with the coordination strength. ^17^O NMR analysis of cyclic ether solvent-based electrolytes revealed systematic variations in oxygen electronic environments linked to solvation capability (Figure 2c and Figure S3). Compared with the pronounced chemical shift of THF and the minimal shift of 1,4-DX, the intermediate shifts observed for 1,3-DX reflected softened Li^+^–solvent interactions, which promote anion-dominated coordination structures. Further evidence from ^1^H NMR (Figure S4) confirmed the structural integrity of 1,3-DX in the electrolyte, with negligible proton signal shifts under LiFSI coordination, underscoring its resistance to solvation-induced conformational changes. This trend aligns with the blue-shifted anion vibrations in Raman spectroscopy, further validating the transition from solvent- to anion-driven solvation, a critical feature for optimizing desolvation kinetics in softly coordinating electrolytes.

The ionic conductivity measurements further corroborated these structural distinctions (Figure 2d and Table S2). LiFSI/THF exhibited the highest ionic conductivity (8.5 mS cm^–1^), followed by LiFSI/1,3-DX (4.8 mS cm^–1^) and LiFSI/1,4-DX (2.8 mS cm^–1^). This hierarchy aligns with the solvent-dependent ion-pairing dynamics: THF’s dominance of CIPs facilitates rapid ion migration, while the increased anion AGGs in dioxanes introduce additional energy barriers for ion transport.

Notably, 1,3-DX demonstrated a unique compromise between the coordination strength and solvation dynamics. The meta-positioned oxygen atoms in 1,3-DX create spatial frustration that simultaneously (i) limits direct solvent coordination and (ii) generates partial desolvation zones favoring FSI^–^ aggregation around Li^+^. Such geometry-induced solvation heterogeneity enables 1,3-DX to achieve anion-dominated coordination networks—a critical feature for stabilizing high-voltage interfaces while maintaining ionic mobility.

While 1,4-DX exhibits weakly solvating characteristics, characterized by minimal interaction with Li^+^, this results in insufficient ion dissociation and, consequently, low ionic conductivity. In contrast, softly solvating solvents are characterized by a combination of low donor numbers and moderately high dielectric constants, which enables them to reduce Li^+^–solvent binding energy without compromising ion transport kinetics. In this work, 1,3-dioxane is selected as a representative softly solvating solvent. It has a donor number comparable to that of 1,4-dioxane but a significantly higher dielectric constant (5.3 for 1,3-DX vs 2.2 for 1,4-DX). This balance allows the 1,3-DX-based electrolyte to achieve significantly higher ionic conductivity under comparable salt concentrations. Electrochemical assessments showed that while LiFSI/1,4-DX demonstrates moderate reversibility and functionality, LiFSI/1,3-DX exhibits superior performance with higher Coulombic efficiency in Li||Cu cells and more stable cycling in Li||NCM523 full cells (Figure S5). Building on that balance as an optimal solvent that balances sufficient ion transport with soft coordination for low interfacial resistance, we strategically integrate LiFSI and LiPF_6_ to address complementary challenges. LiFSI provides high ionic conductivity and interfacial regulation critical for wide-temperature operation,^42–44^ while LiPF_6_ enhances high-voltage stability and suppresses aluminum current collector corrosion.^45,46^ Integrating these findings, we propose a dual-salt electrolyte of 1.35 M LiFSI+0.05 M LiPF_6_ in 1,3-DX (LiFSI+LiPF_6_/1,3-DX) to decouple bulk ion transport and interfacial stabilization through targeted anion functionality. This specific concentration ratio was selected based on systematic optimization, where varying LiFSI/LiPF_6_ ratios were evaluated based on electrochemical performance as shown in Figure S6. The 1.35 M LiFSI concentration is close to its solubility limit in 1,3-DX (∼1.4 M), ensuring complete salt dissolution, while the low LiPF_6_ content effectively enhances the high-voltage stability and suppresses aluminum corrosion without compromising the ionic conductivity. Unlike conventional systems where solvent-dominated solvation limits performance trade-offs, LiFSI in 1,3-DX leverages its highly dissociative FSI^–^ anions to construct stable, inorganic-rich SEI, while LiPF_6_ contributes to enhanced electrochemical stability at elevated voltages. This spatial role differentiation strategy, enabled by the geometric frustration of 1,3-DX that suppresses solvent coordination, allocates FSI^–^ to interfacial engineering (SEI optimization) and PF_6_^–^ to bulk electrolyte stabilization, a synergy unattainable in single-salt configurations: 1.4 M LiFSI in 1,3-DX (LiFSI/1,3-DX) or 1.4 M LiPF_6_ in 1,3-DX (LiPF_6_/1,3-DX). By spatially differentiating anion functionalities (FSI^–^-dominated anode passivation and PF_6_^–^-enhanced cathode stability) rather than relying on solvent–Li^+^ coordination, this design paradigm simultaneously stabilized LMAs and high-voltage cathodes.

### 2.2 Electrochemical Performance and Interphase Stability

The Li||Cu half-cell configuration served as a critical platform for evaluating LMA compatibility and interfacial dynamics with a focus on CE and voltage profile stability. As shown in Figure 3a, the dual-salt electrolyte LiFSI+LiPF_6_/1,3-DX demonstrated exceptional cycling stability with distinct activation behavior. Its initial Coulombic efficiency (ICE) of 49.8% increased to 87.6% in the second cycle as the interface activated, stabilizing at 95.6% from cycle 3 onward. Over 200 cycles (excluding the first two activation cycles), the system achieved an average CE of 98.%, reflecting near-ideal Li reversibility. This performance was accompanied by smooth voltage profiles (Figure 3b) devoid of fluctuations, indicative of stable interfacial kinetics and minimal parasitic reactions. In contrast, LiFSI/1,3-DX exhibited similar initial activation (ICE: 48.3%, cycle 2:89.1%) and marginally higher long-term CE (98.7%). However, its voltage profiles revealed intermittent noise in later cycles, correlating with the porous Li morphology and SEI instability (Figure S7a). Strikingly, LiPF_6_/1,3-DX failed catastrophically, with an average CE of 51.4% over 200 cycles and severe voltage swings, consistent with rampant decomposition and dendritic growth (Figure S7b).

**Figure 3:**
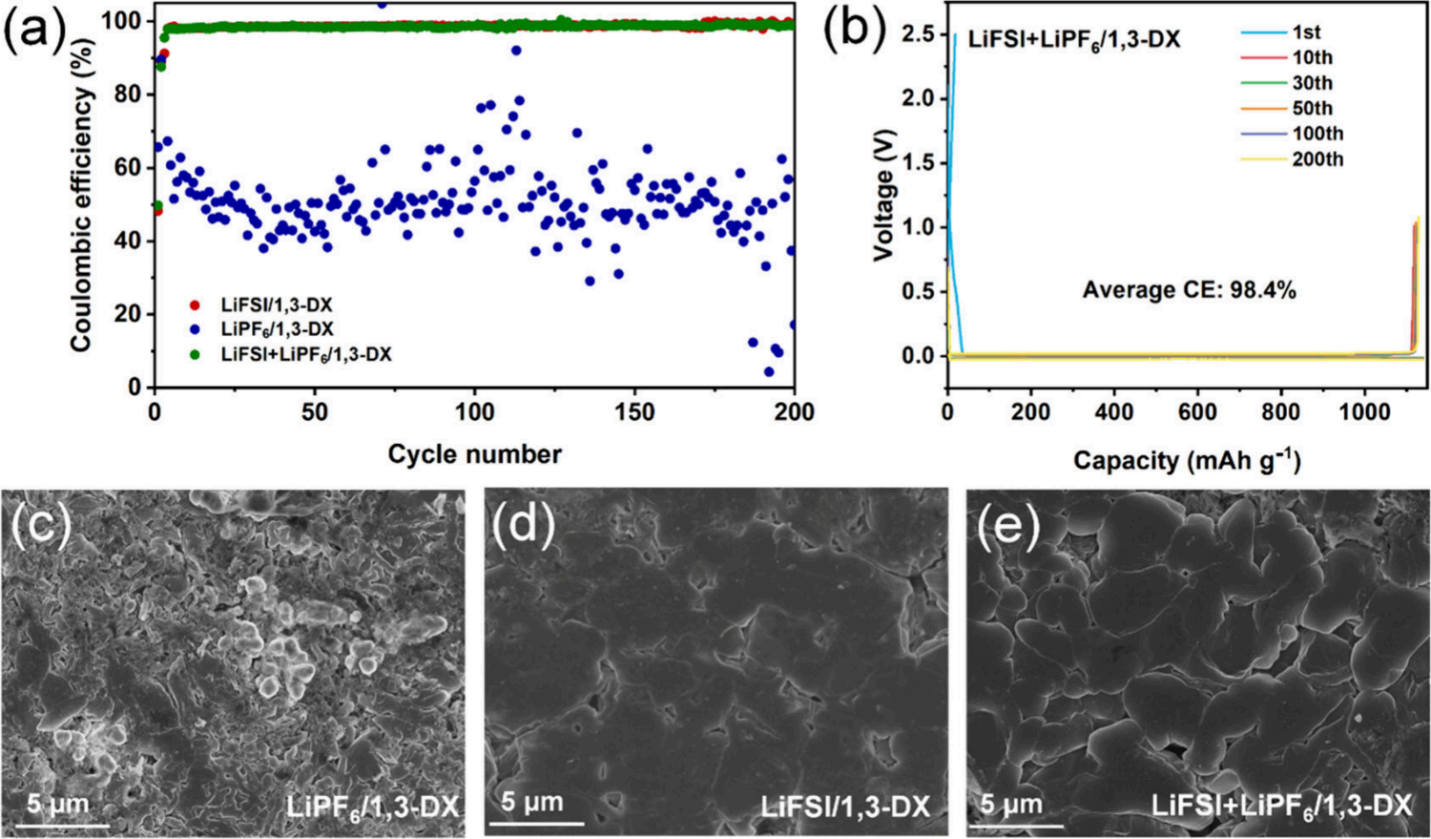
Electrochemical performance and surface morphology of the lithium metal anodes. **a** Coulombic efficiency comparison of three electrolytes over 200 cycles. **b** Voltage–capacity profiles at selected cycles of Li||Cu cells with LiFSI+LiPF_6_/1,3-DX electrolyte. **c**–**e** SEM images of cycled lithium metal anodes in LiPF_6_/1,3-DX, LiFSI/1,3-DX, and LiFSI+LiPF_6_/1,3-DX electrolytes, respectively.

Scanning electron microscopy analysis of LMAs after 15 cycles of Li plating–stripping (Figure 3c–e) revealed distinct morphological trends. Lithium deposition in LiPF_6_/1,3-DX exhibited a porous and loose morphology with extensive voids between particles, a structure that exacerbated interfacial side reactions. Lithium deposition in LiFSI/1,3-DX was relatively uniform but contained localized rough regions, whereas the LiFSI+LiPF_6_/1,3-DX dual-salt electrolyte produced a dense and smooth lithium layer with a thickness of only 8.6 μm (cross-sectional morphology in Figure S8), significantly lower than those in LiFSI/1,3-DX (17.6 μm) and LiPF_6_/1,3-DX (30 μm). These observations suggest that the addition of LiPF_6_ synergized with LiFSI to optimize the SEI composition and mechanical strength, thereby suppressing lithium dendrite growth and volume expansion.

The ionic conductivities of the three electrolytes were measured at 25 °C (Table S3). The LiFSI/1,3-DX system exhibited the highest ionic conductivity (4.8 mS cm^–1^), followed by LiPF_6_/1,3-DX (3.3 mS cm^–1^), while the dual-salt LiFSI+LiPF_6_/1,3-DX showed the lowest value (3.0 mS cm^–1^), and this value remains sufficient for most LMB applications. This reduced ionic conductivity in the LiFSI+LiPF_6_/1,3-DX electrolyte arose from an increased AGG formation. In AGG-dominated electrolytes, ionic diffusion transitions from the “vehicle mechanism” (collective movement of ions with their solvation shells) to the “structural mechanism” (via ion-coupled/decoupled hopping processes), with the latter exhibiting significantly lower mobility. The enhanced AGG content, evidenced by Raman spectral analysis (Figure S9), confirms stronger anion–cation interactions in the dual-salt system, which sacrifices the bulk ion transport efficiency to achieve superior interfacial stability and dendrite suppression. The electrochemical impedance spectroscopy (EIS) of pristine Li||NCM523 cells in Figure S10 revealed a clear hierarchy in interfacial charge-transfer resistance: LiFSI/1,3-DX > LiPF_6_/1,3-DX > LiFSI+LiPF_6_/1,3-DX. The LiFSI+LiPF_6_/1,3-DX electrolyte exhibited the lowest impedance, confirming its superior interfacial kinetics.

In the evaluation of the Li||NCM523 cells tested within a voltage range of 2.7 to 4.3 V, significant differences are shown among various electrolytes. For the LiFSI/1,3-DX electrolyte, despite its superior anode interface stability, the cycling performance was limited. After 0.1 C activation and 0.3 C cycling for 100 cycles, the capacity retention of NCM523 dropped to 48.3% with a CE of 99.0% (Figure 4a). The voltage profiles revealed substantial polarization after 30 cycles (Figure 4b), indicating severe interfacial side reactions and electrolyte oxidation, which deteriorated the electrochemical kinetics. In contrast, the LiPF_6_/1,3-DX electrolyte enabled NMC523 to show improved cathode compatibility (72.5% capacity retention) but suffered from unstable anode interfaces, resulting in a low CE of 94.8%. The capacity underwent a sharp decline after 100 cycles, with the capacity retention rate dropping to merely 18.3% by the 150th cycle, as demonstrated in Figure S11. The pronounced polarization in its voltage profiles further confirmed nonuniform lithium deposition and accumulated interfacial impedance (Figure 4c).

**Figure 4:**
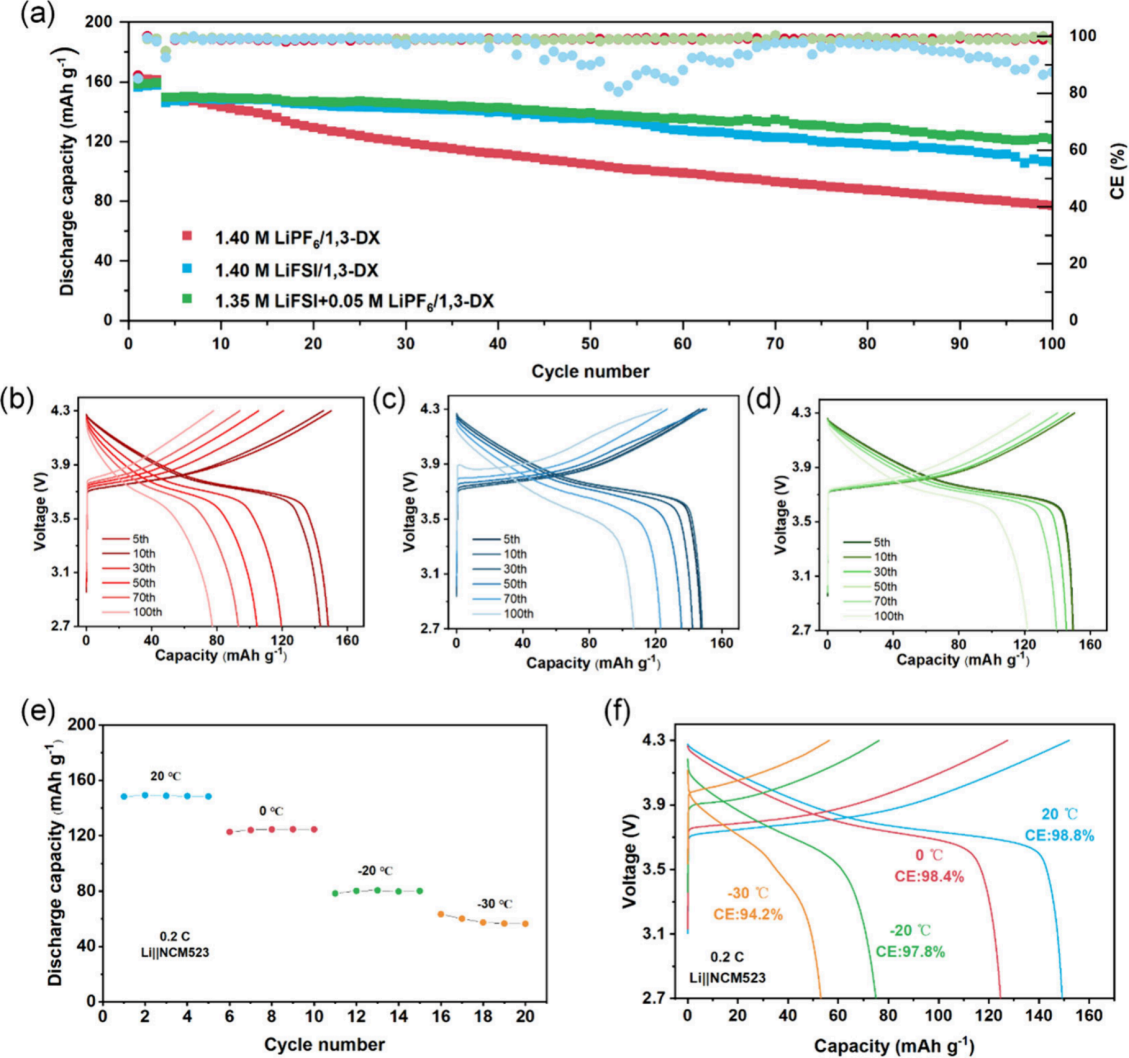
**a** Long-term cycling performance for full cells with three electrolytes. **b** Voltage–capacity profiles of selected cycles for cells with the LiFSI/1,3-DX electrolyte. **c** Voltage–capacity profiles of selected cycles for cells with the LiPF_6_/1,3-DX electrolyte. **d** Voltage–capacity profiles of selected cycles for cells with LiFSI+LiPF_6_/1,3-DX. **e** Discharge capacity of the Li||NCM523 cell with the LiFSI+LiPF_6_/1,3-DX electrolyte at different temperatures under 0.2 C. **f** Voltage–capacity curves for full cells with LiFSI+LiPF_6_/1,3-DX at different temperatures with corresponding Coulombic efficiency values.

The Li||NCM523 cells with a LiFSI+LiPF_6_/1,3-DX dual-salt electrolyte demonstrated optimized cycling performance through synergistic effects. It achieved a capacity retention of 80.3% and a stable CE of 99.0% without noticeable polarization during cycling (Figure 4d). This improvement stemmed from the complementary roles of LiFSI and LiPF_6_: LiFSI facilitated uniform lithium deposition at the anode, while LiPF_6_ enhanced the cathode stability by forming a robust interfacial layer to mitigate electrolyte oxidation under high voltage. Additionally, the LiFSI+LiPF_6_/1,3-DX system optimized lithium-ion transport kinetics by tailoring the solvation structure, thereby maintaining a low interfacial resistance during prolonged cycling. These results highlight that the LiFSI+LiPF_6_/1,3-DX electrolyte design effectively balanced the interfacial requirements of both electrodes, offering a promising solution for high-voltage LMBs.

To systematically assess the wide-temperature compatibility of the LiFSI+LiPF_6_/1,3-DX electrolyte, the low-temperature behavior of Li||NCM523 cells was evaluated through voltage–capacity profiles and cycling stability tests. Figure 4e shows that the cells maintained stable discharge capacities of 148.3–149.3 mAh g^–1^ with little fluctuation during the initial 20 °C. When the temperature was progressively lowered to −20 °C, the Li||NCM523 cells retained capacities of 78.3–80.6 mAh g^–1^, corresponding to 54.1% of their initial capacity at 20 °C. Under extreme (−30 °C) conditions, the capacities stabilized at 56.4–54.7 mAh g^–1^. Notably, all temperature-specific cycling curves exhibit minimal fluctuations and high Coulombic efficiency (as shown in Figure 4f), indicating kinetically stable electrode interfaces across a wide temperature range. These results confirm that the solvation structure reorganization in the LiFSI+LiPF_6_/1,3-DX system effectively mitigates the low-temperature-induced capacity degradation. EIS was conducted to clarify the interfacial properties contributing to the superior low-temperature performance of the LiFSI+LiPF_6_/1,3-DX electrolyte. At 20 °C, the LiFSI+LiPF_6_/1,3-DX electrolyte exhibits the lowest overall impedance among the three tested electrolytes, with an SEI resistance (*R*_SEI_) and charge-transfer resistance (*R*_ct_) of 8.6 and 7.8 Ω, respectively, as shown in Figure S12a and S12b. As the temperature decreases to −20 °C and −30 °C, these resistances in the LiFSI+LiPF_6_/1,3-DX system increase but remain comparatively lower (*R*_SEI_/*R*_ct_: 73.3 Ω/449.0 Ω at −20 °C and 523.0 Ω/1202.0 Ω at −30 °C), indicating better interfacial stability and charge-transfer kinetics at low temperatures (Figure S12c and S12d).

The rate performance in Figure S13 shows that the LiFSI+LiPF_6_/1,3-DX electrolyte delivered balanced performance, particularly in exhibiting excellent rate capability up to 3 C and full capacity recovery upon returning to 1 C. This highlights the synergistic benefits for enhancing ionic transport and interfacial stability.

Transmission electron microscope analysis reveals significant differences in the thickness of the interfacial layer of three electrolytes on the NCM523 cathode as illustrated in Figure 5a–c. Distinct interfacial architectures were observed across the three electrolyte formulations. The thickness of the CEI for the LiFSI/1,3-DX electrolyte was formed around 17.8 nm. This layer exhibited an irregular morphology characterized by local thickness variations and surface roughness, which indicated nonuniform interfacial reactions occurring during cycling. In contrast, the CEI layer for the LiPF_6_/1,3-DX electrolyte thickened to 20.1 nm. This layer displayed moderate surface undulations and fluctuations in layer continuity, suggesting a more stable formation process compared with LiFSI. For the LiFSI+LiPF_6_/1,3-DX electrolyte, a notably thin CEI of only 6.1 nm with a uniform morphology was observed. This indicated an optimized interfacial reaction process, which might enhance the battery performance by minimizing resistance at the interface and improving ionic conductivity.

**Figure 5:**
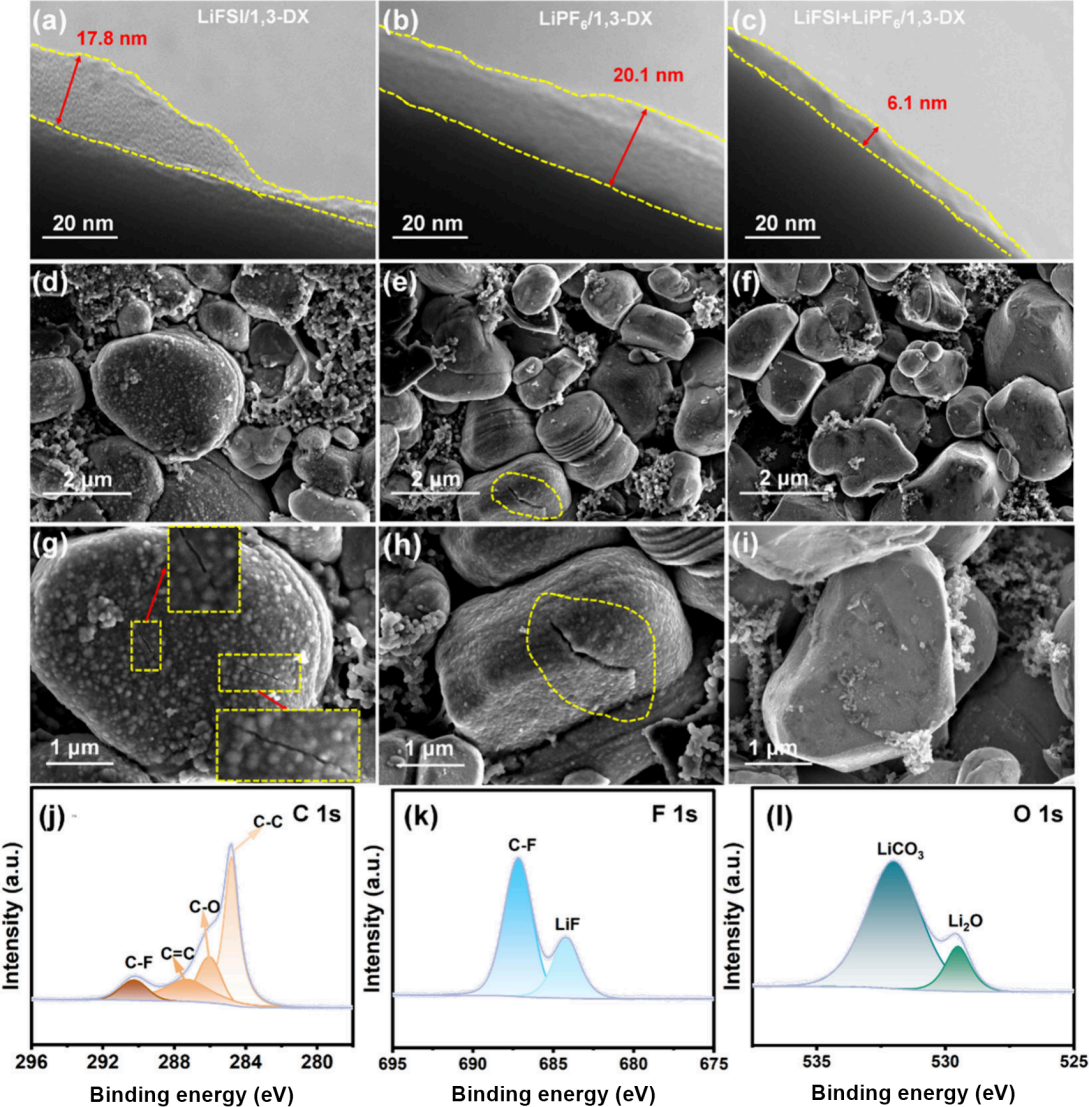
Multiscale characterization of NCM523 cathode cycling in various electrolytes. **a**–**c** TEM images of the CEI formed in LiFSI/1,3-DX, LiPF_6_/1,3-DX, and LiFSI+LiPF_6_/1,3-DX electrolytes. **d**–**i** SEM images showing the surface morphology of cycled NCM523 cathodes under different electrolytes. **j**–**l** XPS deconvoluted spectra of C 1s, F 1s, and O 1s recorded for the LiFSI+LiPF_6_/1,3-DX electrolyte.

The SEM analysis of cycled NCM523 cathodes revealed distinct interfacial degradation patterns across electrolyte systems. In the LiFSI/1,3-DX electrolyte, pronounced intergranular cracks were observed, propagating along particle boundaries and indicative of localized mechanical stress accumulation during cycling (Figure S14). These cracks likely stem from inhomogeneous interfacial reactions and insufficient passivation. By contrast, cycled NCM523 cathodes in the LiPF_6_/1,3-DX and LiFSI+LiPF_6_/1,3-DX electrolytes exhibited comparable surface morphologies, both displaying uniform particle arrangements without significant cracking or structural discontinuities, but critical distinctions emerged upon closer inspection. In the LiPF_6_/1,3-DX electrolyte (Figure 5e and h), extensive intragranular cracks traversed individual particles, forming fracture networks indicative of severe mechanical degradation. Similarly, the LiFSI/1,3-DX system (Figure 5d and g) revealed microcracks and surface pitting, though to a lesser extent than those observed in the LiPF_6_/1,3-DX electrolyte. In stark contrast, the LiFSI+LiPF_6_/1,3-DX dual-salt electrolyte (Figure 5f and i) demonstrated exceptional structural preservation, with NCM523 particles retaining smooth surfaces and minimal defects even under high-resolution imaging. The absence of cracking in this system highlighted the synergistic role of FSI^–^ and PF_6_^–^ anions in stabilizing interfacial reactions, effectively mitigating stress accumulation during Li^+^ cycling. This nanoscale structural homogeneity aligned with the enhanced electrochemical performance of LiFSI+LiPF_6_/1,3-DX electrolytes, underscoring their potential for high-voltage battery applications. To further substantiate the “crack-free” structural integrity observed on the particle surfaces, cross-sectional SEM characterization of cycled NCM523 particles was conducted (Figure S15). The images confirm that particles cycled in the LiFSI+LiPF_6_/1,3-DX electrolyte maintain their internal structure without visible cracks or fractures, consistent with the surface observations in Figure 5f and 5i. Additionally, inductively coupled plasma mass spectrometry (ICP-MS) quantification of the electrolyte after cycling revealed a significant reduction in transition metal dissolution—specifically Ni, Co, and Mn—in cells using the LiFSI+LiPF_6_/1,3-DX electrolyte compared to single-salt counterparts (Figure S16). This suppression of the metal ion highlights the formation of a robust and effective CEI in the LiFSI+LiPF_6_/1,3-DX system.

X-ray photoelectron spectroscopy (XPS) analysis (Figure 5j–l) revealed the presence of LiF, Li_2_O, and carbonaceous species across all electrolyte systems. The XPS peak intensities and binding energies of cycled electrodes using LiFSI/1,3-DX and LiPF_6_/1,3-DX electrolytes are presented in Figure S17. While these components were universally present in the CEIs, the superior morphological integrity and electrochemical performance of the LiFSI+LiPF_6_/1,3-DX system likely originated primarily from its structural homogeneity, as evidenced by the absence of intragranular cracking rather than from unique compositional features. Linear sweep voltammetry (LSV) in Li||Al cells (Figure S18) demonstrated that the LiFSI+LiPF_6_/1,3-DX dual-salt electrolyte exhibited an extended electrochemical stability window compared to that of LiFSI/1,3-DX and with significantly suppressed decomposition currents at high voltages.

While the boosted electrochemical performance originates from the DX-based solvation design, practical implementation requires addressing inherent safety considerations. To enhance the electrolyte’s flame resistance, different proportions of TTE were mixed with 1,3-DX. Specifically, the electrolyte with a 75:25 volume ratio of 1,3-DX to TTE was used to assemble Li||NCM523 cells, which exhibited a capacity retention of 78.1% after 100 cycles at a high cutoff voltage of 4.3 V, as shown in Figure S19. Moreover, the dual-salt formulation inherently mitigates stability concerns; the strategic limitation of LiPF_6_ to 0.05 M suppresses its decomposition pathways while retaining its cathode-stabilizing function.

## 3 Conclusion

In this work, we designed a softly solvating dual-salt electrolyte using 1,3-dioxane, which weakens the Li^+^–solvent binding strength by asymmetric oxygen geometry. Raman spectroscopy revealed that solvent geometric frustration promotes anion-aggregate coordination, surpassing the inherent instability of single-salt-derived interphases. By synergization of LiFSI and LiPF_6_, the LiFSI+LiPF_6_/1,3-DX electrolyte enables cross-electrode stabilization: LiFSI promotes robust SEI formation at the lithium metal anode, while LiPF_6_ facilitates stable CEI formation at the cathode through preferential anion decomposition. This complementary interfacial mechanism enables 4.3 V Li||NCM523 cells to retain 80% of their initial capacity after 100 cycles, outperforming both LiFSI and LiPF_6_ single-salt systems. At −30 °C, the Li||NCM523 cells maintain 36.9% capacity retention (54.7 mAh g^–1^) relative to room-temperature capacity (148.3 mAh g^–1^), validating effective ion transport under low-temperature conditions. Overall, this work addresses the long-standing challenge of dual-electrode compatibility in high-voltage LMBs and demonstrates that salt synergy, combined with solvent geometry engineering, offers a powerful strategy for spatially selective interfacial modulation.

## 4 Experimental Section

### 4.1 Materials

The lithium metal foil was bought from China Energy Lithium Co., Ltd., while the solvents, including tetrahydrofuran (THF), 1,3-dioxane (1,3-DX), and 1,4-dioxane (1,4-DX), were procured from Aladdin Biochemical Technology Co., Ltd. The LiNi_0.5_Co_0.2_Mn_0.3_O_2_ (NCM523) cathodes, lithium bis­(fluorosulfonyl)­imide (LiFSI), and lithium hexafluorophosphate (LiPF_6_), all battery-grade, were supplied by Canrd Co., Ltd.

### 4.2 Electrochemical Testing Protocols

The electrolytes were prepared by dissolving 1.0 M LiFSI in pure solvents under vigorous magnetic stirring within an argon-filled glovebox ([H_2_O] < 0.1 ppm, [O_2_] < 0.1 ppm). Prior to cell assembly, the NCM523 electrodes (areal capacity: 1.63 mAh cm^–2^, loadings around 10.5 mg cm^–2^) were vacuum-dried at 80 °C for 12 h to eliminate residual moisture and subsequently punched into 12 mm diameter disks. Coin cells (CR2032) were assembled using lithium metal anodes paired with NCM523 cathodes or copper foil, separated by Celgard 2325 membranes. The electrolyte volume was precisely controlled at 40 μL for Li||NCM523 cells and 60 μL for Li||Cu cells via a micropipette. The Li||NCM523 coin cells were cycled between 2.7 and 4.3 V (vs Li/Li^+^). All experiments were conducted at 25 °C unless otherwise specified.

### 4.3 Characterization Methods

The cycled electrodes were characterized by using a Hitachi SU-8010 field-emission scanning electron microscope (SEM). Chemical composition analysis of electrode interfaces was performed via X-ray photoelectron spectroscopy (XPS) on a Shimadzu Axis Supra^+^ spectrometer (Al–Kα source, 1486.6 eV), with binding energies referenced to carbon C 1s (284.8 eV). Structural evolution of cycled NCM523 cathode particles was observed by transmission electron microscopy (TEM) on a Hitachi HT-7700. Raman spectra were acquired by using a Renishaw inVia system with a 633 nm laser. Nuclear magnetic resonance (NMR) spectroscopy (^1^H and ^17^O) was conducted on a Bruker DPX 400 spectrometer (600 MHz) with deuterium lock using D_2_O as the locking solvent. The ionic conductivity of electrolytes was measured via electrochemical impedance spectroscopy (EIS) in stainless steel symmetric cells (SS|electrolyte|SS, CR2032). Each electrolyte was tested in triplicate at 25 °C.

### 4.4 Computational Calculations

The calculations were conducted by the Gaussian 16 package, and the structures of molecules were optimized with the B3LYP/6-311+G­(d, p) level of theory.^47^ Visualizations of the electrostatic potential (ESP) were produced through color-filled isosurface graphs created with the Visual Molecular Dynamics (VMD) software.^48^ The binding energy was calculated as follows:

$$E_{b}=E_{total}-E_{\mathrm{sol}}-E_{\mathrm{Li}}$$

*E*_Li_, *E*_sol_, and *E*_total_ represent the energy of Li^+^, solvent, and Li^+^–solvent coordination.

## Supplementary Material



## Notes

Spectroscopic characterization, electrochemical data, microstructural analysis, supplementary tables (PDF)
