# Supplementary material for: Anion-Complementary Soft Solvation Electrolytes Stabilizing Dual Interfaces for High-Voltage Lithium Metal Batteries across Wide Temperatures
Source: ACS Appl Mater Interfaces. 2025 Sep 18;17(39):54623–32. doi: 10.1021/acsami.5c07417 (PMC12755193; doi:10.1021/acsami.5c07417)
Supplement: Supplementary file 1 [file am5c07417_si_001.pdf]

Supporting information

Anion-Complementary Soft Solvation Electrolytes Stabilizing Dual Interfaces for High-Voltage Lithium Metal Batteries Across Wide Temperatures

Siyu Sun[1,2#], Huipeng Zeng[2#], Baichuan Cui[2], Mingjia Zhi[1], Jing Zheng[3], Zhanglian Hong[1*], Jijian Xu[2, 4*]*

1 State Key Laboratory of Silicon Materials, School of Materials Science and Engineering, Zhejiang University, Hangzhou, 310027 P. R. China.

2 Department of Chemistry, City University of Hong Kong, Hong Kong 999077, China.

3 Department of Chemistry and Materials Science, College of Science, Nanjing Forestry University, Nanjing 210037, P.R. China

[4] Shenzhen Research Institute, City University of Hong Kong, Shenzhen, 518057, China

*Corresponding author. E-mail: jzheng62@njfu.edu.cn; hong_zhanglian@zju.edu.cn; jijianxu@cityu.edu.hk

# These authors contributed equally

Keywords, Lithium metal batteries, Ether-based electrolyte, Electrolyte design, Solid Electrolyte Interphase, Wide-temperature

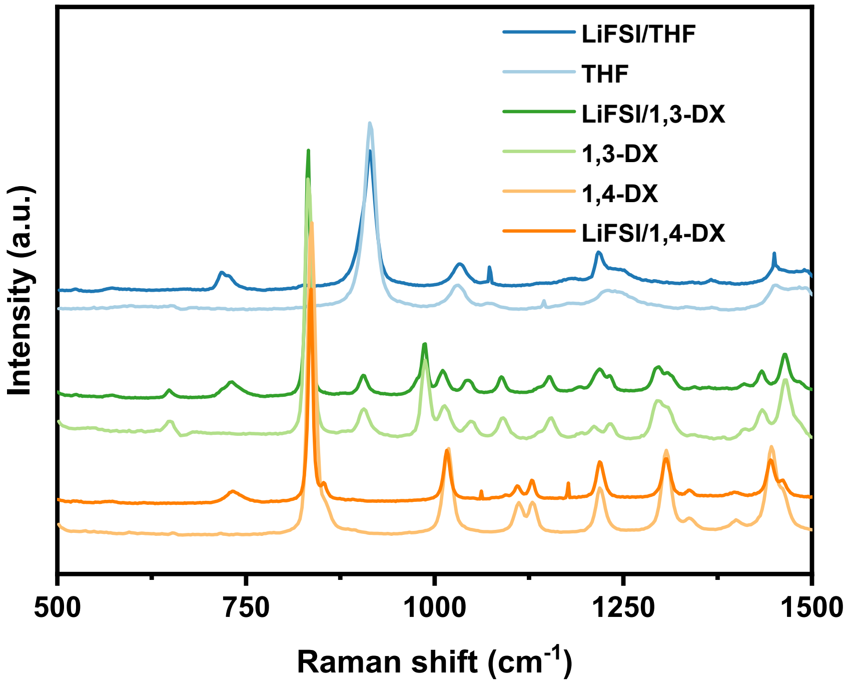

Figure S1. Full Raman spectra of various LiFSI-based electrolytes using different solvents.

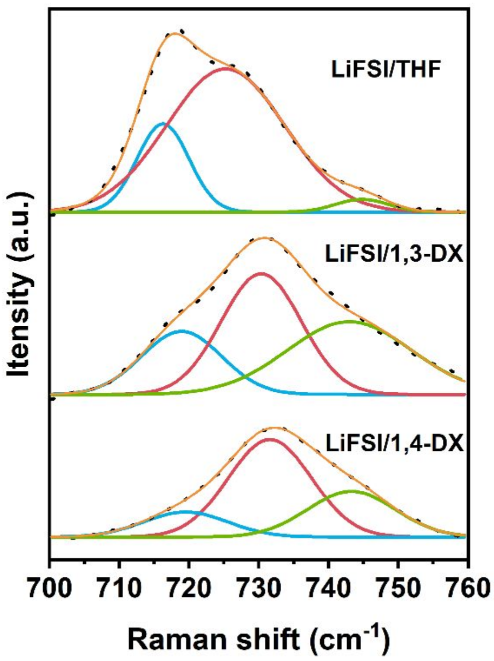

Figure S2. Raman spectra of FSI^{-} vibrations in 1.4 M LiFSI electrolytes with different cyclic ether solvents.

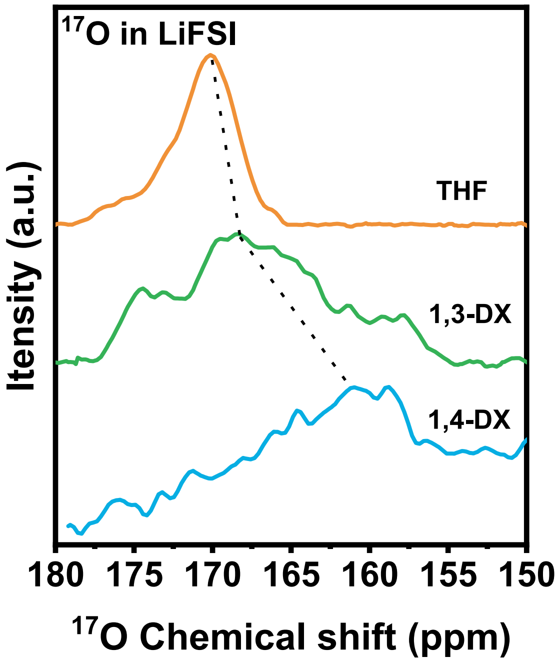

Figure S3. ^{17}O NMR spectra of three electrolytes.

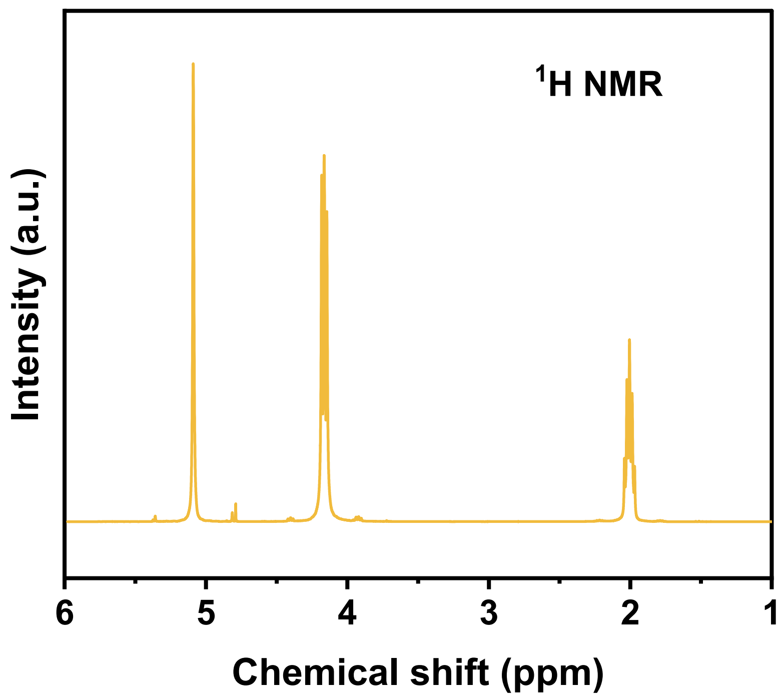

Figure S4. ^{1}H NMR spectra of 1.4 M LiFSI/1,3-DX electrolyte.

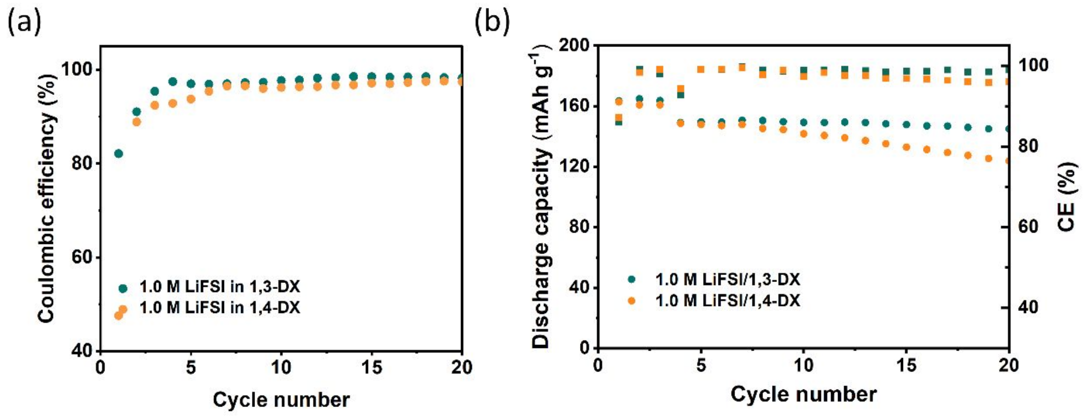

Figure S5. Comparison of electrochemical performance between 1.0 M LiFSI in 1,3-DX and 1.0 M LiFSI in 1,4-DX electrolytes. (a) Coulombic efficiency of Li||Cu. (b) Cycling performance of Li||NCM523.

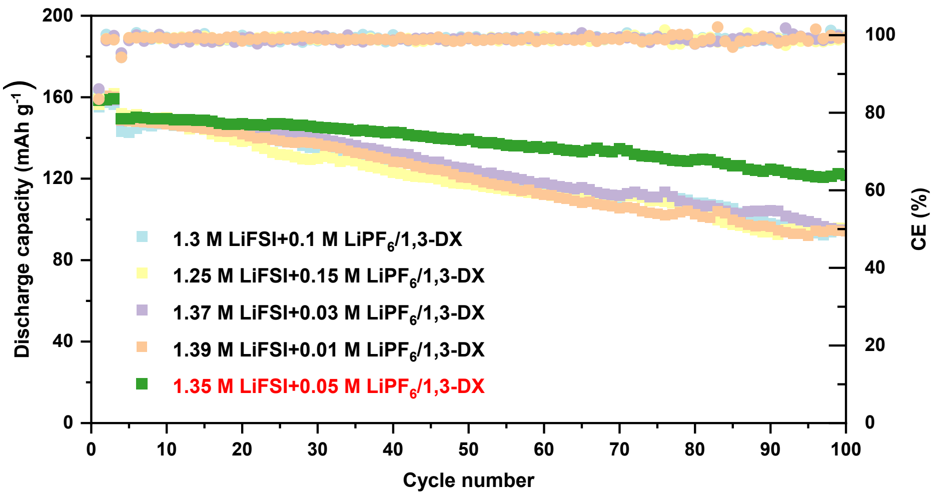

Figure S6. Cycling performance of NCM523 cathodes in different electrolytes: 1.3 M LiFSI+0.1 M $LiPF_6$, 1.25 M LiFSI+0.15 M $LiPF_6$, 1.37 M LiFSI+0.03 M $LiPF_6$, 1.39 M LiFSI+0.01 M $LiPF_6$, 1.35 M LiFSI+0.05 M $LiPF_6$.

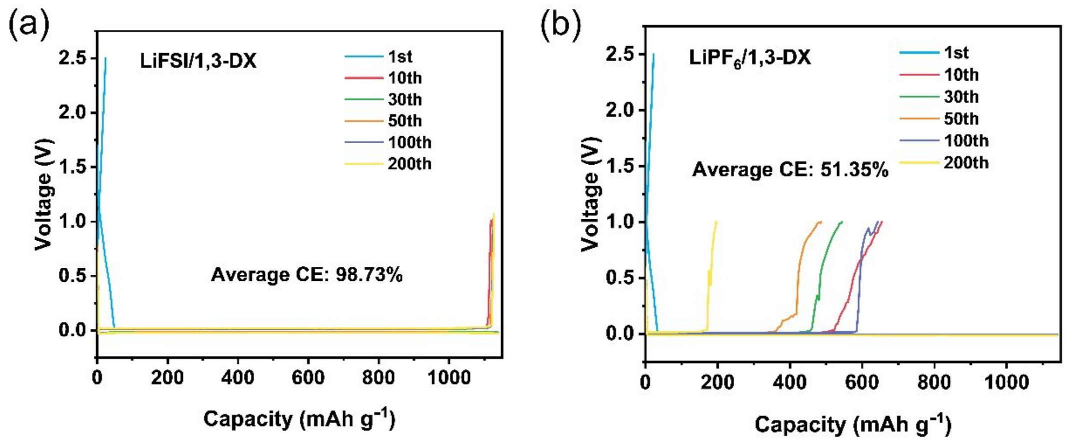

Figure S7. Voltage-capacity profiles at selected cycles for (a) LiFSI/1,3-DX and (b) $LiPF_6$/1,3-DX electrolytes.

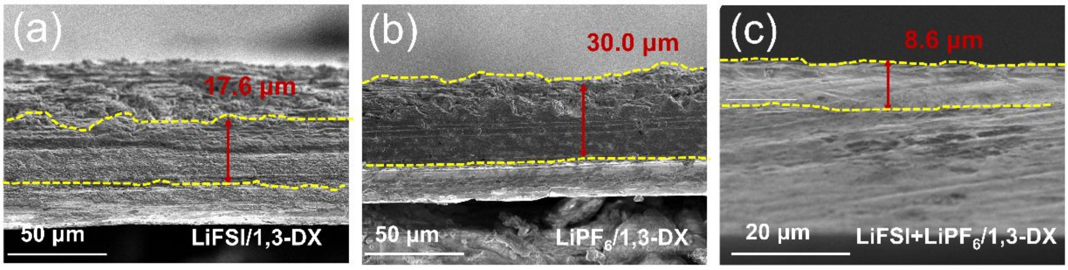

Figure S8. Cross-sectional SEM images of electrode interfaces. (a) LiFSI/1,3-DX; (b)$LiPF_6$/1,3-DX; (c) LiFSI+$LiPF_6$/1,3-DX.

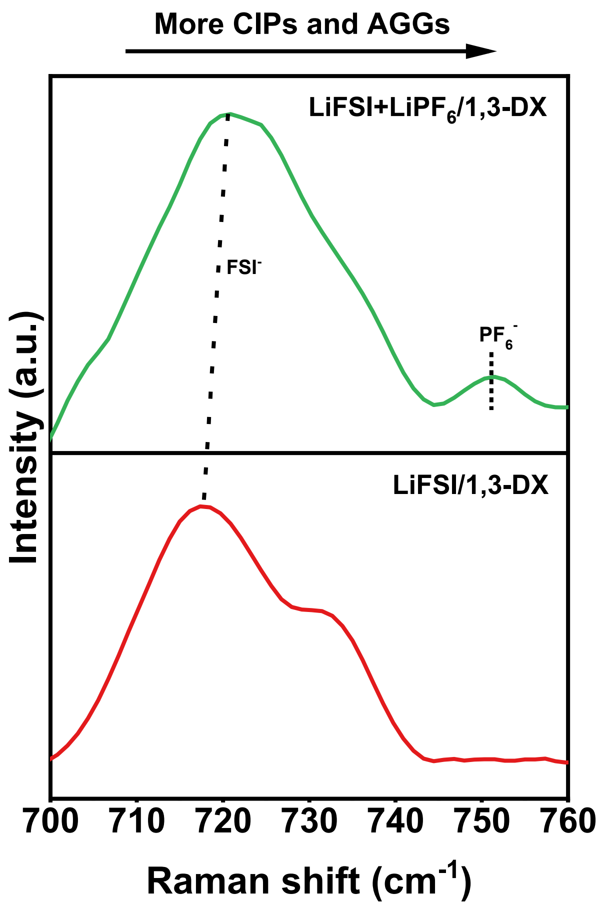

Figure S9. Raman spectra of single-salt electrolyte of LiFSI/1,3-DX and dual-salt electrolyte of $LiFSI+LiPF_6$/1,3-DX within the range of 700-760 cm^{-1}.

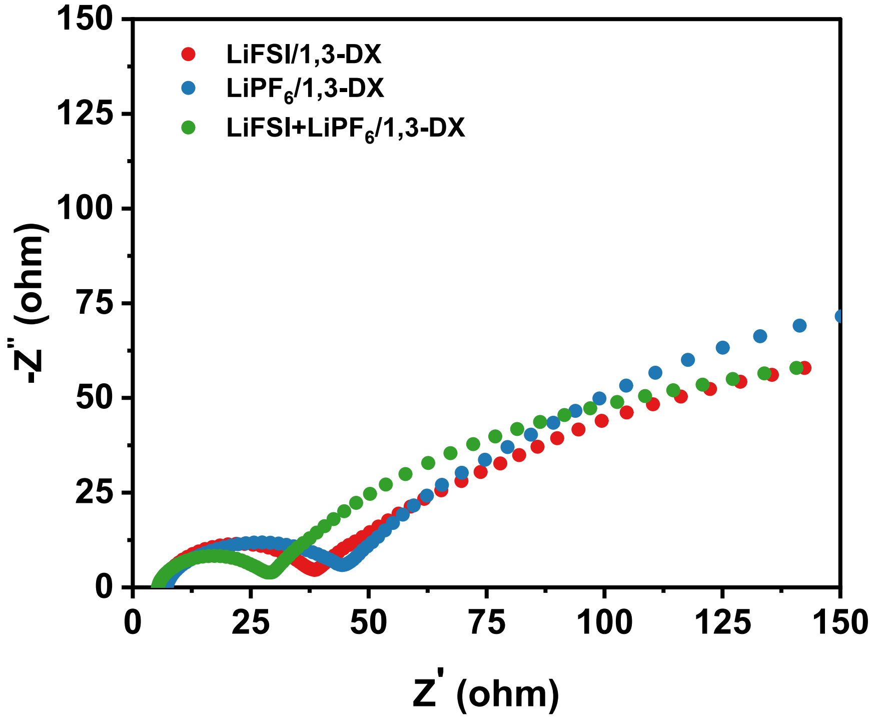

Figure S10. EIS of different electrolytes based on 1,3-DX solvent at room temperature.

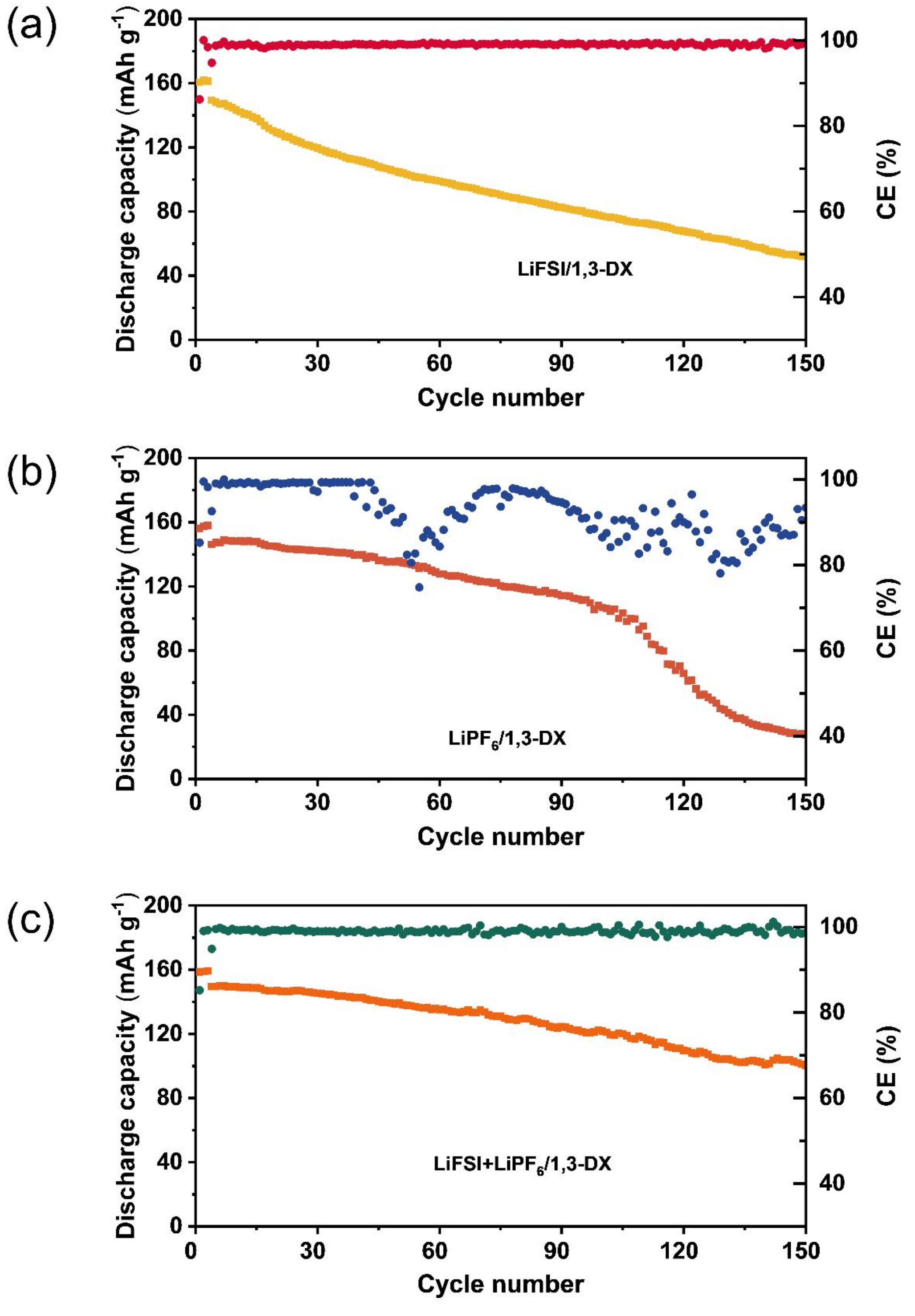

Figure S11. Cycling performance of NCM523 cathodes in different electrolytes.

(a) LiFSI/1,3-DX; (b)$LiPF_6$/1,3-DX; (c) LiFSI+$LiPF_6$/1,3-DX.

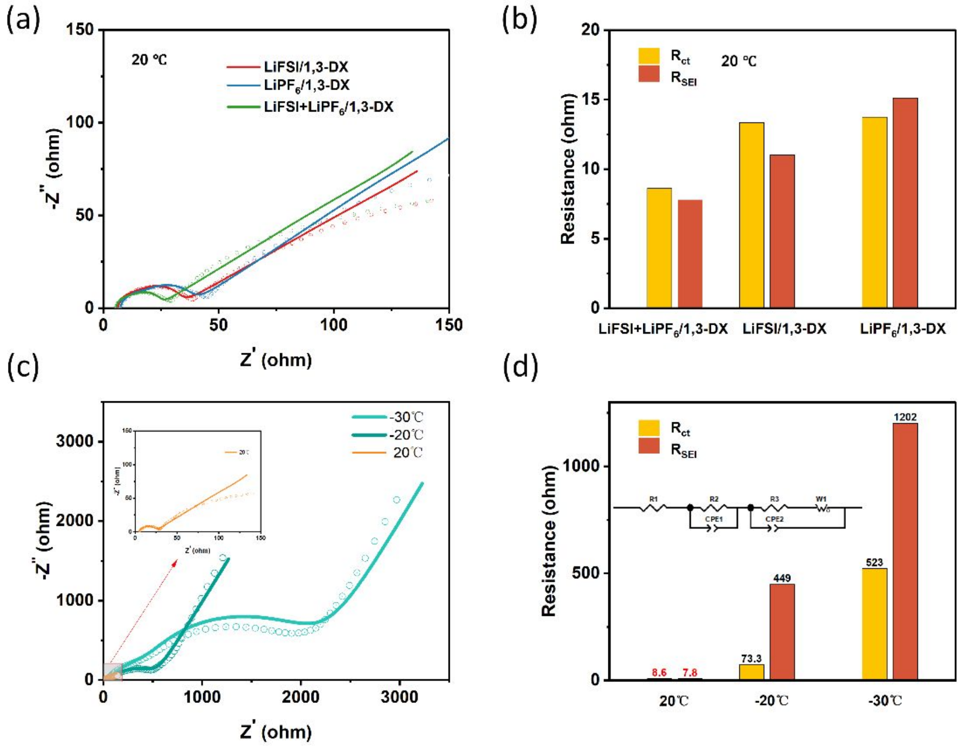

Figure S12. (a) EIS of LiFSI/1,3-DX, LiPF6/1,3-DX, and LiFSI+LiPF6/1,3-DX electrolytes at room temperature; (b) Corresponding SEI resistance (RSEI) and charge-transfer resistance (Rct); (c) EIS of LiFSI+LiPF6/1,3-DX electrolyte at 20 °C, −20 °C, and −30 °C; (d) Corresponding RSEI and Rct at different temperatures.

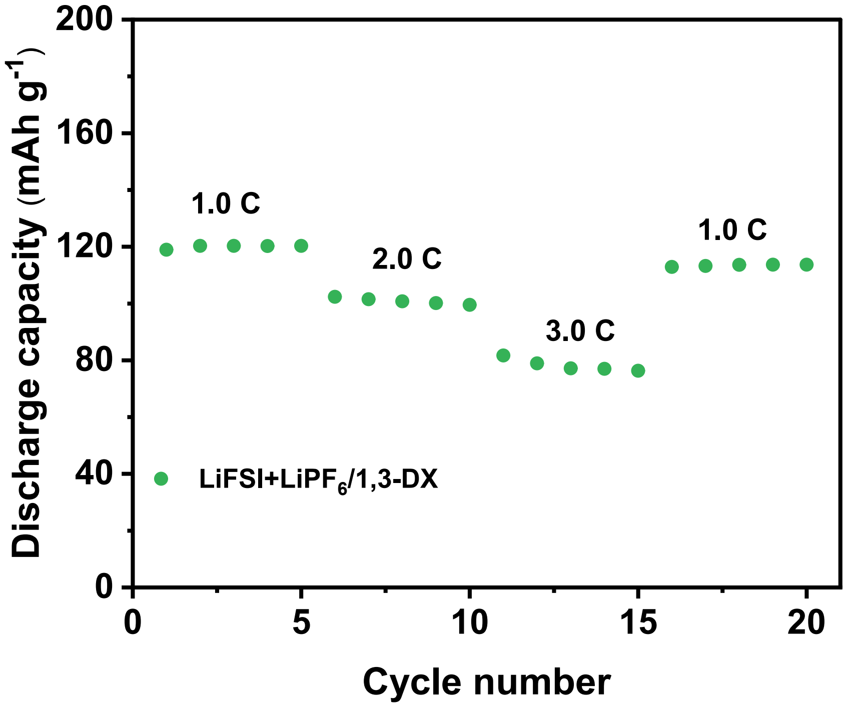

Figure S13. Discharge capacity of Li||NCM523 in LiFSI+$LiPF_6$/1,3-DX under various charge and discharge rates.

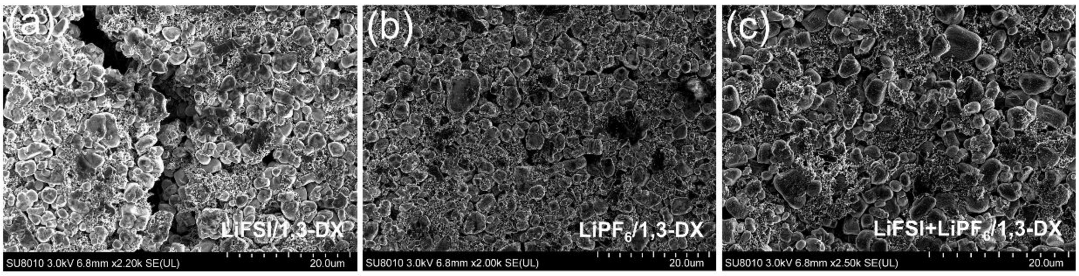

Figure S14. Surface morphology of cycled NCM523 cathodes. (a) LiFSI/1,3-DX; (b)$LiPF_6$/1,3-DX; (c) LiFSI+$LiPF_6$/1,3-DX.

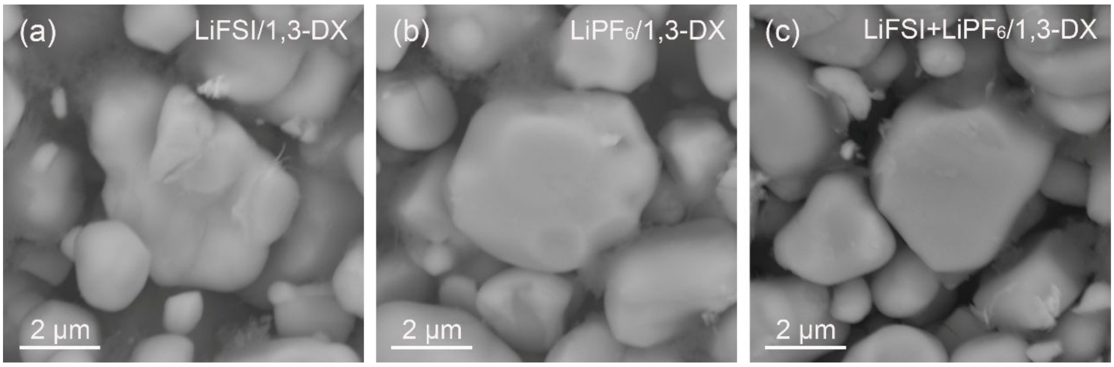

Figure S15. Surface morphology of cycled NCM523 cathodes. (a) LiFSI/1,3-DX; (b)$LiPF_6$/1,3-DX; (c) LiFSI+$LiPF_6$/1,3-DX.

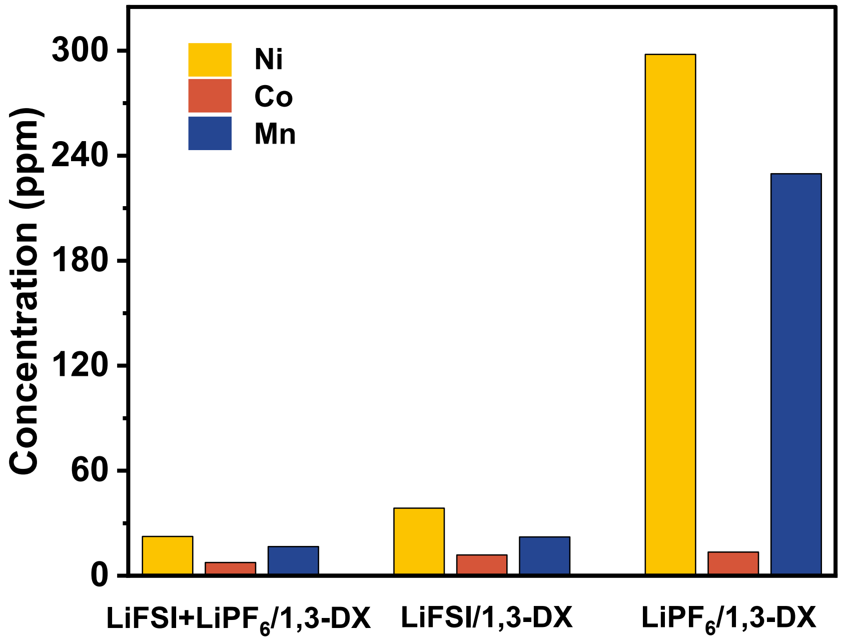

Figure S16. The content of transition metals Ni, Co, and Mn in LiFSI/1,3-DX, $LiPF_6$/1,3-DX, and LiFSI+$LiPF_6$/1,3-DX electrolytes after 10 cycles.

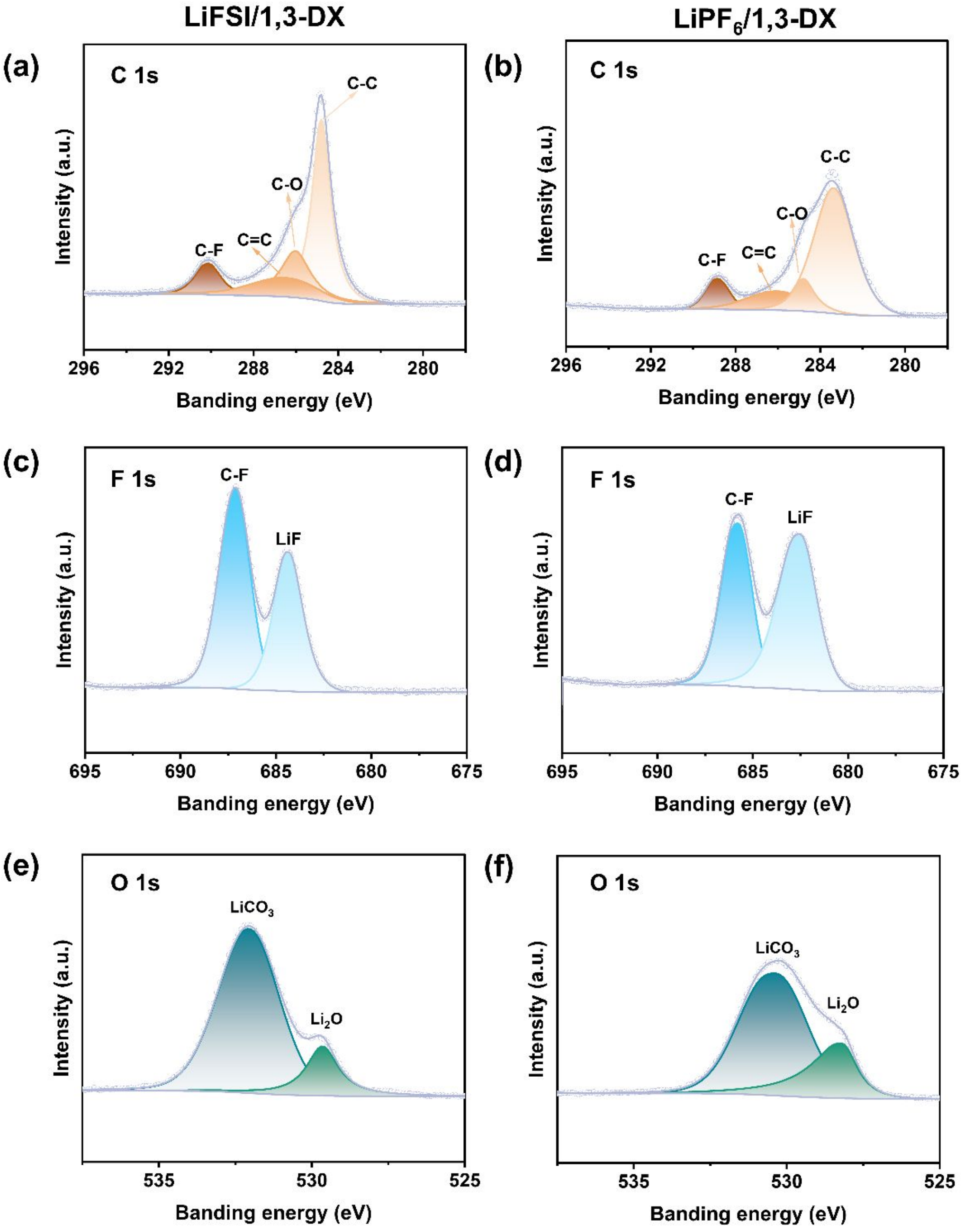

Figure S17. XPS spectra of cycled electrodes using LiFSI/1,3-DX and $LiPF_6$/1,3-DX electrolytes. LiFSI/1,3-DX: (a) C 1s, (c) F 1s, (e) O 1s. $LiPF_6$/1,3-DX: (b) C 1s, (d) F 1s, (f) O 1s.

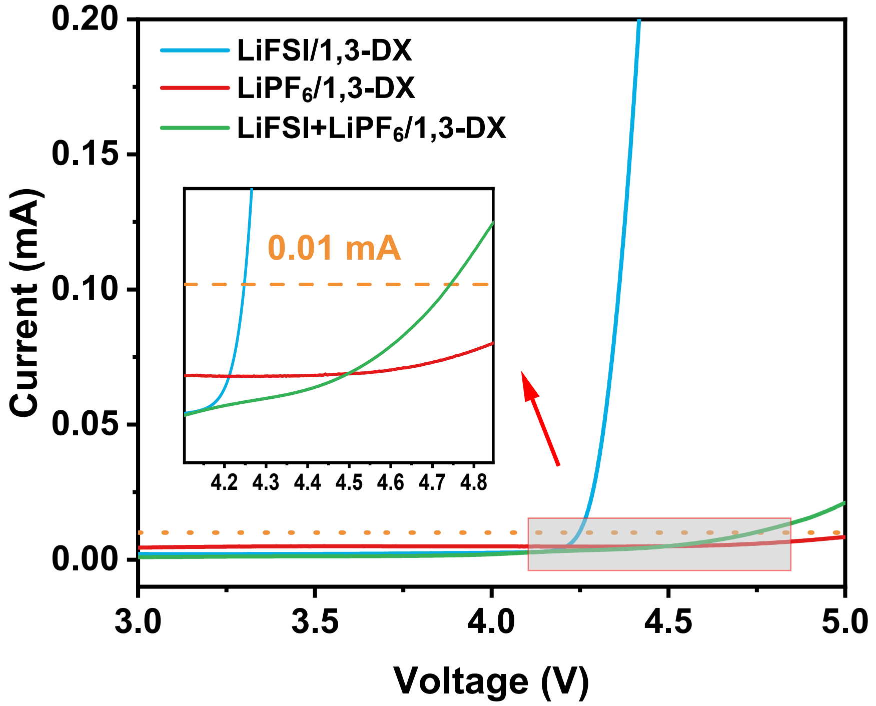

Figure S18. LSV profiles of 1,3-DX-based electrolytes at a scan rate of 1 mV/s.

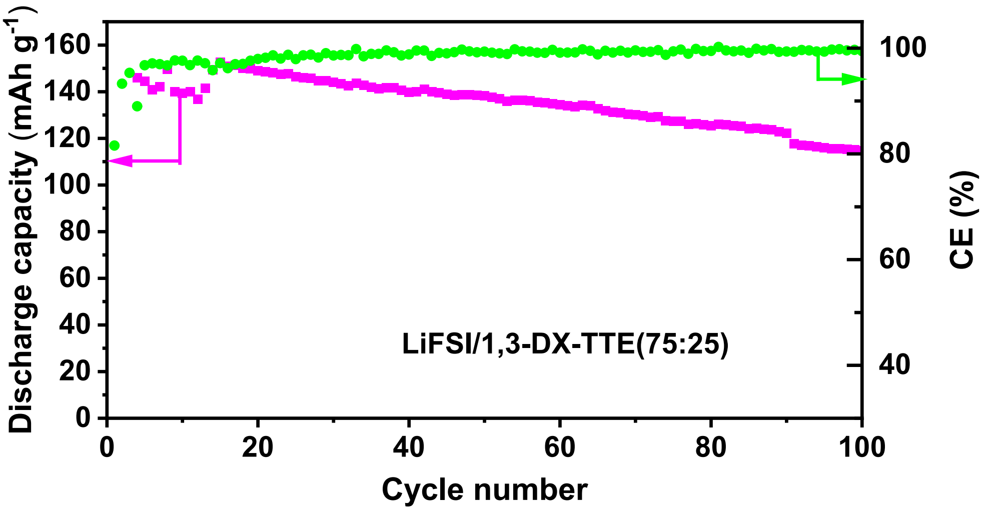

Figure S19. Cycling performance of NCM523 cathodes in LiFSI/1,3-DX-TTE(75:25).

Table S1. Intrinsic properties of THF, 1,4-DX and 1,3-DX solvents.

Solvent	Boiling Point	Melting Point	Density	Dielectric Constant
THF	66.0 °C	−108.38 °C	0.8833 g cm^{-3}	7.5
1,4-DX	101.2 °C	11.75°C	1.0337 g cm^{-3}	2.2
1,3-DX	105.0 °C	−45.00 °C	1.0286 g cm^{-3}	5.3

Table S2. Ionic conductivity of LiFSI-based electrolytes at the same concentration in 1,4-DX, 1,3-DX, and THF.

Electrolyte	Ionic conductivity (mS cm^{-1})
1.4 M LiFSI/1,4-DX	2.8
1.4 M LiFSI/1,3-DX	4.8
1.4 M LiFSI/THF	8.5

Table S3. Ionic conductivity of three electrolytes based on 1,3-DX.

Electrolyte	Ionic conductivity (mS cm^{-1})
LiFSI/1,3-DX	4.8
$LiPF_6$/1,3-DX	3.3
LiFSI+$LiPF_6$/1,3-DX	3.0
